# Supplementary material for: pH and Pectinase Dual-Responsive Zinc Oxide Core-Shell Nanopesticide: Efficient Control of Sclerotinia Disease and Reduction of Environmental Risks
Source: Nanomaterials (Basel). 2024 Dec 16;14(24):2022. doi: 10.3390/nano14242022 (PMC11728501; doi:10.3390/nano14242022)
Supplement: Supplementary file 1 [file nanomaterials-14-02022-s001.zip › nanomaterials-3322893-supplementary.pdf]

Supplementary Materials

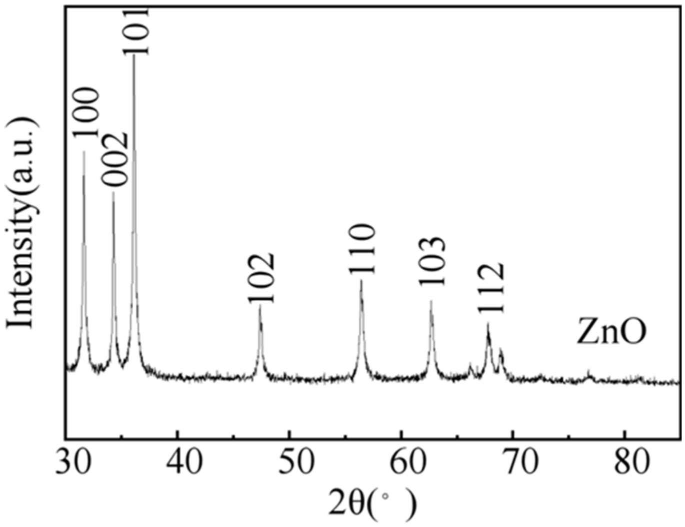

Figure S1. XRD pattern of ZnO, basically consistent with the peak values of the experiment in this paper [1].

References

1. Liu, Y.; Hu, Y.; Zhou, M.; Qian, H.; Hu, X. Microwave-assisted non-aqueous route to deposit well-dispersed ZnO nanocrystals on reduced graphene oxide sheets with improved photoactivity for the decolorization of dyes under visible light. *Appl. Catal. B Environ.* **2012**, *125*, 425–431.
